# Epigenetic regulation of BAF60A determines efficiency of miniature swine iPSC generation

**DOI:** 10.1038/s41598-022-12919-6

**Published:** 2022-05-31

**Authors:** Hongli Jiao, Ming-Song Lee, Athillesh Sivapatham, Ellen M. Leiferman, Wan-Ju Li

**Affiliations:** 1 Laboratory of Musculoskeletal Biology and Regenerative Medicine, Department of Orthopedics and Rehabilitation, University of Wisconsin-Madison, 1111 Highland Ave, WIMR 5051, Madison, WI 53705 USA; 2 Department of Biomedical Engineering, University of Wisconsin-Madison, Madison, WI USA

**Keywords:** Cell biology, Stem cells

## Abstract

Miniature pigs are an ideal animal model for translational research to evaluate stem cell therapies and regenerative applications. While the derivation of induced pluripotent stem cells (iPSCs) from miniature pigs has been demonstrated, there is still a lack of a reliable method to generate and maintain miniature pig iPSCs. In this study, we derived iPSCs from fibroblasts of Wisconsin miniature swine (WMS), Yucatan miniature swine (YMS), and Göttingen minipigs (GM) using our culture medium. By comparing cells of the different pig breeds, we found that YMS fibroblasts were more efficiently reprogrammed into iPSCs, forming colonies with well-defined borders, than WMS and GM fibroblasts. We also demonstrated that YMS iPSC lines with a normal pig karyotype gave rise to cells of the three germ layers in vitro and in vivo. Mesenchymal stromal cells expressing phenotypic characteristics were derived from established iPSC lines as an example of potential applications. In addition, we found that the expression level of the switch/sucrose nonfermentable component BAF60A regulated by STAT3 signaling determined the efficiency of pig iPSC generation. The findings of this study provide insight into the underlying mechanism controlling the reprogramming efficiency of miniature pig cells to develop a viable strategy to enhance the generation of iPSCs for biomedical research.

## Introduction

Pigs sharing anatomical, physiological, and functional features of tissues and organs with humans are ideal as animal models for biomedical research. For example, with remarkable similarity to the human epidermis, porcine skin has been used to study and develop treatments for injuries^1–3^. In addition, there is emerging interest in developing transgenic pigs for disease modeling and generating humanized pigs for xenotransplantation of organs^4–6^. In regenerative medicine, pigs are considered a viable translational model to validate in vitro or small animal studies before human clinical trials^7^.

Cellular reprogramming of pig somatic cells to generate induced pluripotent stem cells (iPSCs) was first demonstrated more than a decade ago following the derivation of mouse and human iPSCs^8^. To date, several studies using different reprogramming approaches to generate pig iPSCs have been reported. For example, a study used retroviral pMX vectors containing mouse *Oct4*, *Sox2*, *Klf4*, and *c-Myc* to generate iPSC lines from fetal fibroblasts of Tibetan miniature pigs^9^. Another study derived iPSCs from fetal fibroblasts through lentiviral transduction of human *OCT4*, *SOX2*, *KLF4*, and *c-MYC*^10^. It has been shown that the four Yamanaka factors together with *Nanog* and *Lin28* are used to induce the generation of pig iPSCs^11^. The iPSC lines produced in these studies are considered primed pluripotent stem cells, lacking the capacity to generate chimeric pigs. To derive naive pig iPSCs, several groups have optimized induction medium by supplementing leukemia inhibitory factor (LIF) and inhibitors of epigenetic regulators^12–14^. Nevertheless, it remains challenging to generate pig iPSCs with the desired efficiency following previously published protocols. The reprogramming efficiency of pig cells is relatively low compared to that of human or mouse cells, regardless of whether integrating or nonintegrating approaches are used^10,15–17^.

Reprogramming somatic cells into iPSCs requires chromatin remodeling; thus, regulating chromatin remodeling can control the efficiency of cellular reprogramming^18^. Epigenetic enzymes, including the switch/sucrose nonfermentable (SWI/SNF) complex, are involved in remodeling chromatin structure, modifying nucleosome position, and regulating gene transcription in mammalian cells^19–22^. During cellular reprogramming, BAF components of the SWI/SNF family facilitate the binding of exogenously introduced Oct4 to promoters of key pluripotency regulators, such as *Oct4*, *Nanog*, *Rex1*, and *Fbx15*, to upregulate their expression^23^. In addition, the expression of SWI/SNF family proteins is strongly correlated with iPSC reprogramming efficiency. A recent study has demonstrated that the reprogramming efficiency of dermal fibroblasts from African American donors is higher than that from European American donors, resulting from differential regulation of SWI/SNF protein activities^24^. This finding suggests a potential strategy to enhance the reprogramming efficiency of pig iPSCs by modulating the activity of the SWI/SNF chromatin remodeling complex.

In this study, we compared the efficiency of iPSC generation among three different breeds of miniature pigs. We chose Wisconsin miniature swine (WMS), Yucatan miniature swine (YMS), and Göttingen minipigs (GM) because they are commonly used for research and are also available for our study. We generated and characterized iPSCs derived from fibroblasts of these breeds using nonviral cellular reprogramming and further determined the molecular cause of the difference in the efficiency of iPSC generation.

## Results

### Reprogramming efficiency varies among different breeds of miniature pigs

**Figure 1:**
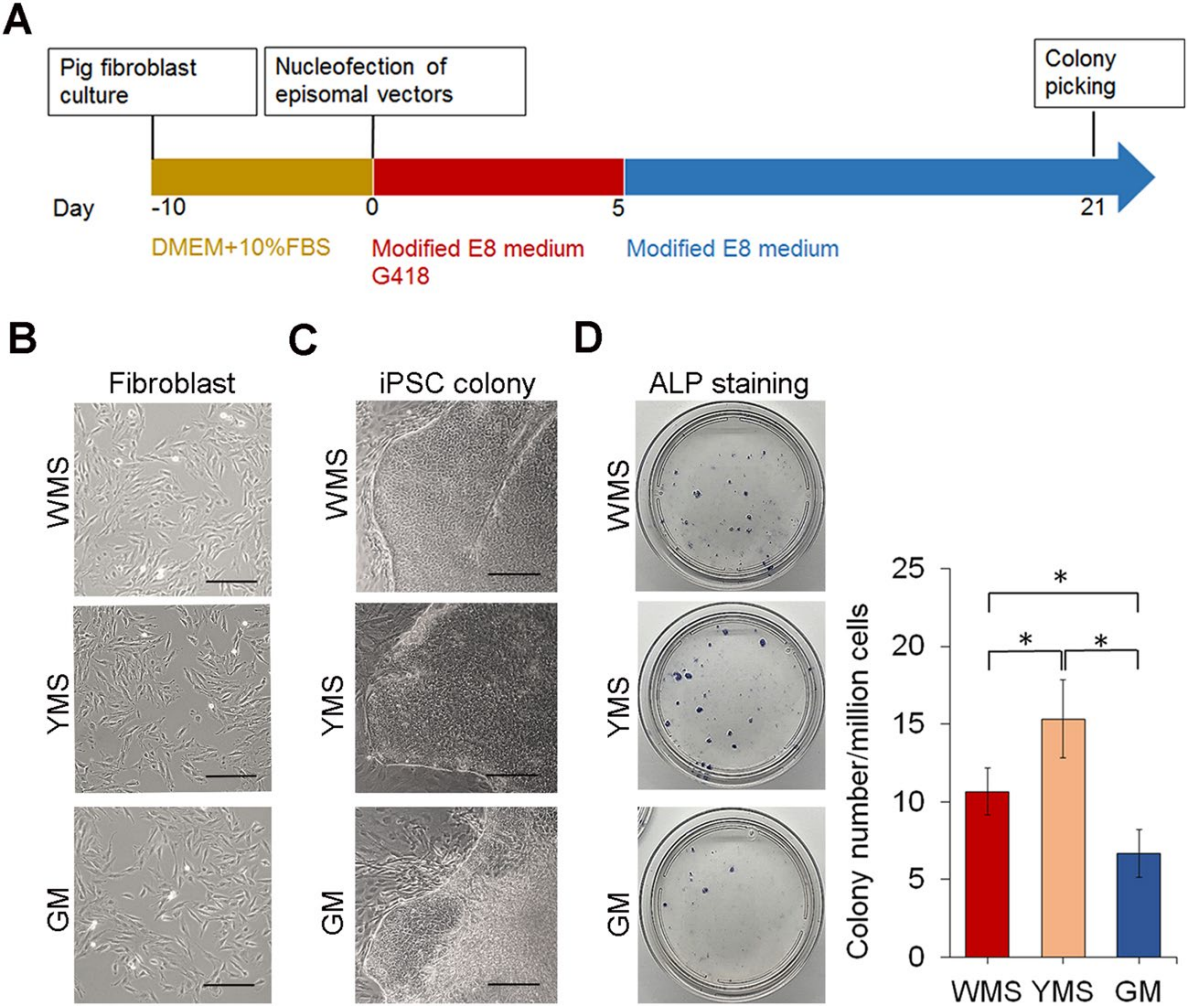
Generation of iPSCs from fibroblasts of three breeds of miniature pigs. (**A**) Schematic diagram illustrating the timeline and stepwise procedures to establish iPSC lines. (**B**) Phase-contrast images showing flat, elongated cells with typical fibroblast morphology. (**C**) Phase-contrast images showing pig iPSC colonies with well-defined borders at day 21 post transfection. (**D**) ALP staining of iPSC colonies and corresponding reprogramming efficiency presented by the number of ALP-positive colonies per million cells. ALP-positive colonies in each 100-mm dish were counted. Scale bar = 200 µm. *p < 0.05; n = 3.

We generated iPSC lines from WMS, YMS, and GM fibroblasts by transfecting episomal vectors pMaster12^17^ into 3 independent fibroblast lines of each of the pig breeds and inducing them stepwise with different culture compositions (Fig. 1A). Parental fibroblasts of the three breeds exhibited similar spindle-shaped morphology (Fig. 1B). Embryonic stem cell (ESC)-like colonies with well-defined borders and comprised of small, tightly packed cells with large nuclei appeared approximately 21 days after transfection (Fig. 1C). The counts of alkaline phosphatase (ALP)-positive iPSC colonies derived from 1 million fibroblasts of WMS, YMS, or GM 21 days post-transfection were 10.7 ± 1.5, 15.3 ± 2.5, or 6.7 ± 1.5, respectively (Fig. 1D), indicating that YMS fibroblasts were reprogrammed more effectively into iPSCs than WMS or GM fibroblasts.

### iPSCs derived from fibroblasts of miniature pigs possess pluripotent characteristics

**Figure 2:**
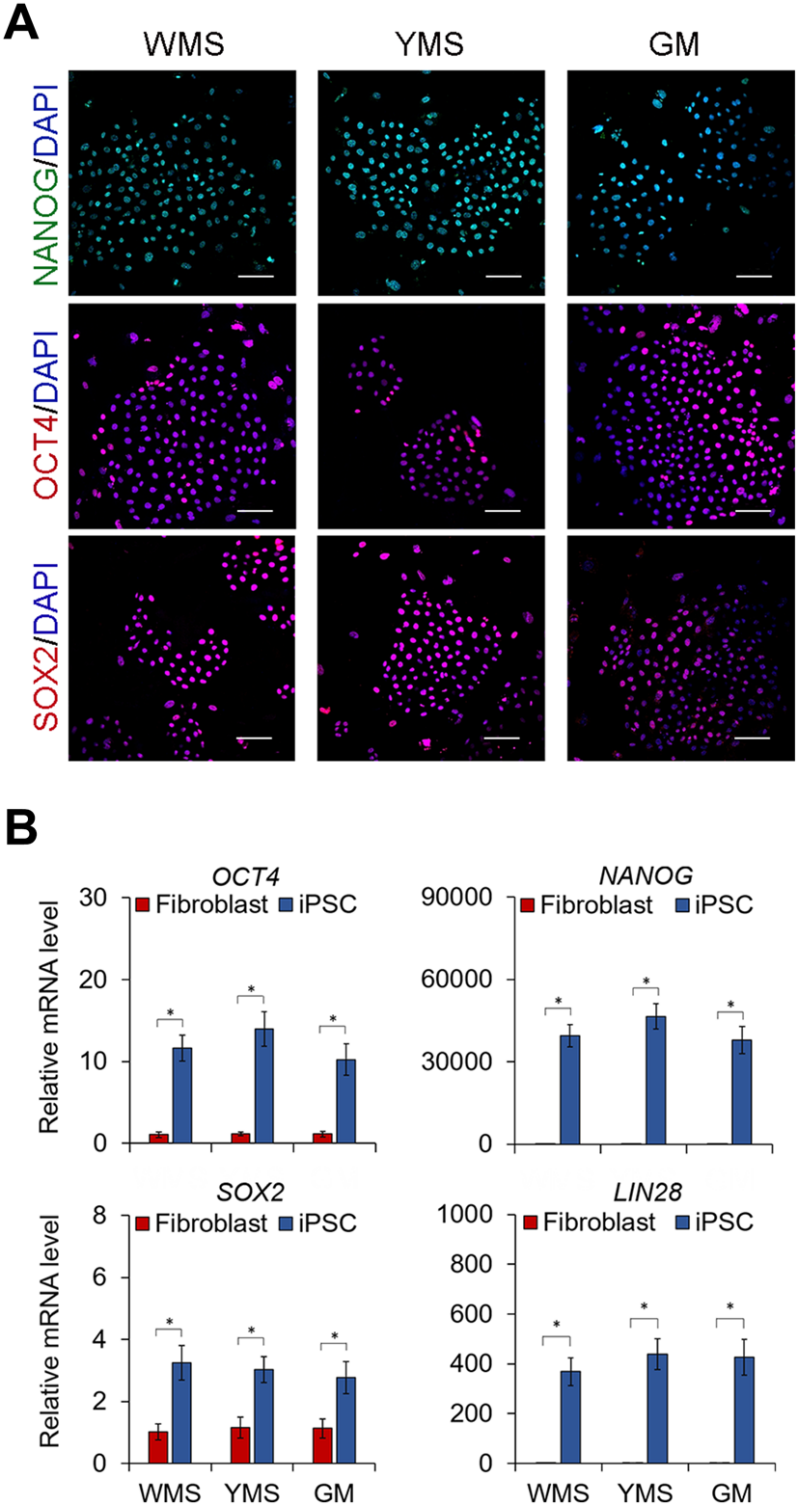
Expression of pluripotency markers in pig iPSCs and parental fibroblasts. (**A**) Immunofluorescence staining detecting the expression of the pluripotency markers NANOG, OCT4, and SOX2 in iPSC lines generated from WMS, YMS, and GM fibroblasts. DAPI stains nuclei (blue). (**B**) Relative mRNA levels of the pluripotency markers *NANOG, OCT4, SOX2,* and *LIN28* determined by quantitative RT–PCR. Scale bar = 100 µm. *p < 0.05; n = 3.

**Figure 3:**
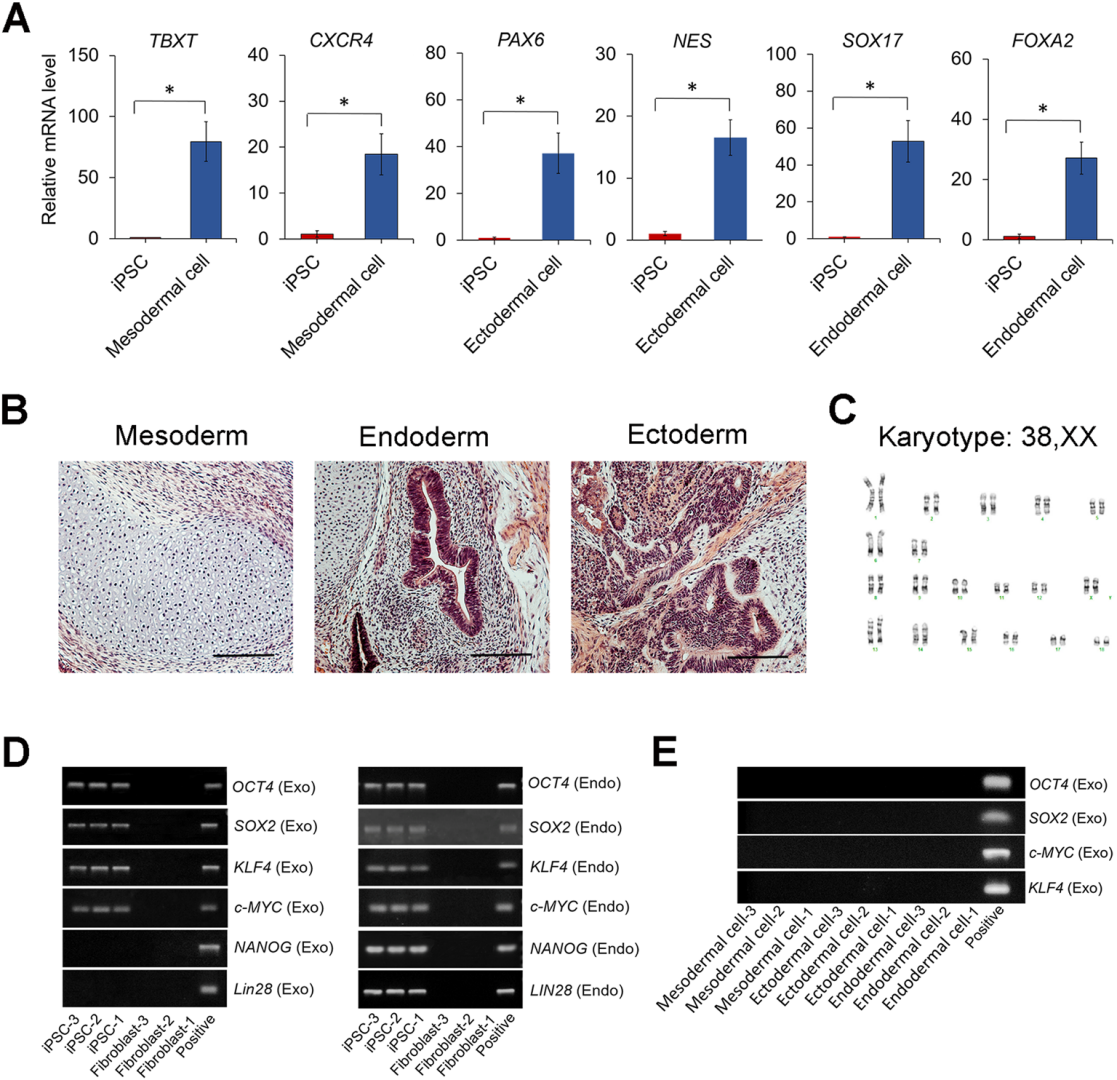
Derivation of 3 germ layer cells and karyotype of pig iPSCs. (**A**) Quantitative RT–PCR detecting relative mRNA levels of the germ layer-associated markers *TBXT* and *CXCR4* (mesoderm), *PAX6* and *NES* (ectoderm), and *SOX17* and *FOXA2* (endoderm) in cells derived from iPSCs after 7 days of germ layer-specific induction. (**B**) Hematoxylin and eosin (H&E) staining of teratomas derived from a representative pig iPSC line. Tissues of 3 germ layers, including cartilage (mesoderm), gut-like epithelium (endoderm), and neuronal rosette (ectoderm), were present in a teratoma. (**C**) Chromosome analysis of a representative iPSC line revealing a normal diploid pig cell with a 38, XX karyotype. (**D**) Expression of exogenous and endogenous pluripotency markers in 3 fibroblast lines (p5) and 3 iPSC lines (p15). (**E**) Expression of exogenous Yamanaka factors in mesodermal, ectodermal, and endodermal cells derived from iPSCs. Original gels are presented in Supplementary Fig. S4. Scale bar = 200 µm. *p < 0.05; n = 3.

Colonies were picked manually on day 21 and expanded to establish iPSC lines. The established iPSC lines of each breed detected by immunofluorescence staining uniformly expressed NANOG, OCT4, and SOX2 (Fig. 2A). Quantitative reverse transcription–polymerase chain reaction **(**RT–PCR) analysis demonstrated that the expression of *OCT4*, *SOX2*, *NANOG, and LIN28* was significantly upregulated after reprogramming (Fig. 2B). Based on the reprogramming efficiency results shown in Fig. 1D, we then selected YMS iPSC lines for further characterization. To assess the pluripotency of YMS iPSCs, germ layer differentiation was induced in culture for 7 days. Transcriptional levels of the mesoderm-specific markers T-box transcription factor T (*TBXT*) and C-X-C motif chemokine receptor 4 (*CXCR4*), ectoderm-specific markers paired box 6 (*PAX6*) and nestin *(NES)*, and endoderm-specific markers SRY-box 17 (*SOX17*) and forkhead box A2 (*FOXA2*) were significantly increased in differentiated cells compared to those in undifferentiated iPSCs (Fig. 3A). Furthermore, the iPSC lines implanted in immunodeficient mice developed teratomas comprised of mesodermal, endodermal, and ectodermal tissues, where cartilage, gastro-intestine-like structure, and neuronal rosette, respectively, were identified (Fig. 3B). The results of cytogenetic analysis indicated that the iPSCs maintained a normal karyotype of miniature pig cells (Fig. 3C).

To determine if the iPSC lines generated through the episomal reprogramming method were free of transgenes, we analyzed the expression of both endogenous and exogenous pluripotency markers. We found that while endogenous pluripotency markers were robustly expressed, exogenous Yamanaka factors remained in iPSCs at passage 15 (Fig. 3D). This unexpected finding suggests that due to some unknown causes, these iPSC lines continued to express exogenous Yamanaka factors. However, once the iPSCs differentiated into mesodermal, ectodermal, or endodermal lineage cells, the four exogenous factors were no longer expressed in the derivatives (Fig. 3E).

### iPSCs give rise to mesenchymal stromal cells (MSCs) with multilineage differentiation potential

**Figure 4:**
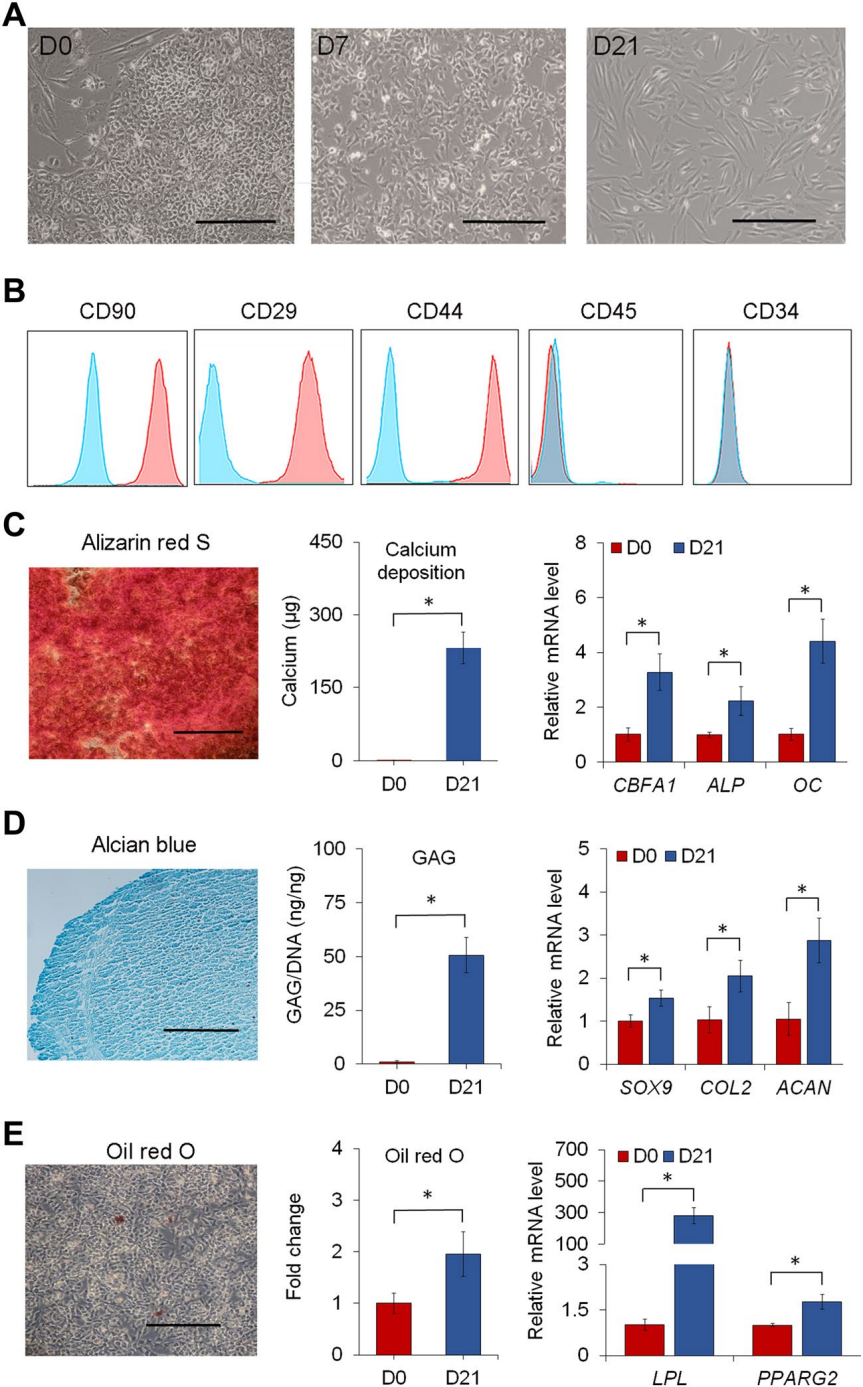
Derivation and characterization of iPSC-MSCs. (**A**) Phase-contrast images of cells in iPSC culture induced for 21 days of MSC differentiation. (**B**) Expression of surface markers on iPSC-MSCs. Pink histograms represent surface markers of interest. Blue histograms represent isotype controls. (**C**) Alizarin red S staining, quantification of calcium deposition, and relative mRNA levels of the bone-associated markers *CBFA1*, *ALP,* and *OC* in cells differentiated from iPSC-MSCs after 21 days of osteogenesis. (**D**) Alcian blue staining, quantification of GAG production, and relative mRNA levels of the cartilage-associated markers *SOX9*, *COL2*, and *ACAN* in cells differentiated from iPSC-MSCs after 21 days of chondrogenesis. (**E**) Oil red O staining, quantification of lipid droplets, and relative mRNA levels of the fat-associated markers *LPL* and *PPARG2* in cells differentiated from iPSC-MSCs after 21 days of adipogenesis. Scale bar = 200 µm. *p < 0.05; n = 3.

To demonstrate the potential of miniature pig iPSCs for regenerative applications, YMS iPSCs were induced to differentiate into MSCs to establish iPSC-MSC lines. After 21 days of induction, iPSC-MSCs exhibited uniform, fibroblast-like morphology (Fig. 4A). We then determined whether iPSC-MSCs possessed MSC characteristics by analyzing the expression of the MSC surface markers CD90, CD29, and CD44 and the hematopoietic stem cell markers CD45 and CD34. As shown in Fig. 4B, the results of flow cytometric analysis revealed that iPSC-MSCs expressed CD90, CD29, and CD44 but not CD45 and CD34. The expression of exogenous pluripotency markers was not found in iPSC-MSCs (Supplementary Fig. S1), and unlike parental iPSCs giving rise to teratomas, iPSC-MSCs did not result in tumor formation (data not shown). We further determined the multilineage differentiation potential of iPSC-MSCs. When induced for lineage-specific differentiation in culture, iPSC-MSCs underwent osteogenesis, chondrogenesis, or adipogenesis. For osteogenesis, calcium deposition was found in culture after 21 days of induction (Fig. 4C). Transcript levels of the bone-related markers core-binding factor subunit alpha-1 (*CBFA1*), *ALP*, and osteocalcin (*OC*) were upregulated at day 21 of induction compared to those at day 0. After 21 days of chondrogenic induction, cell pellets of iPSC-MSCs were stained positive for Alcian blue and produced an increased amount of glycosaminoglycans (GAGs) (Fig. 4D). Quantitative RT–PCR analysis showed that iPSC-MSC pellets expressed significantly higher transcript levels of the cartilage-associated markers sex determining region Y-box 9 (*SOX9*), collagen type 2 (*COL2*), and aggrecan (*ACAN*) at day 21 than those at day 0. For adipogenesis, iPSC-MSCs produced lipid droplets after 21 days of adipogenic induction (Fig. 4E). Transcript levels of the fat-associated markers peroxisome proliferator-activated receptor gamma 2 (*PPARG2*) and lipoprotein lipase (*LPL*) in iPSC-MSCs increased at day 21 compared to those at day 0. These results together suggest that MSCs can be derived from miniature pig iPSCs for regenerative applications.

### Telomere length and DNA methylation are altered in response to cellular reprogramming and MSC differentiation

**Figure 5:**
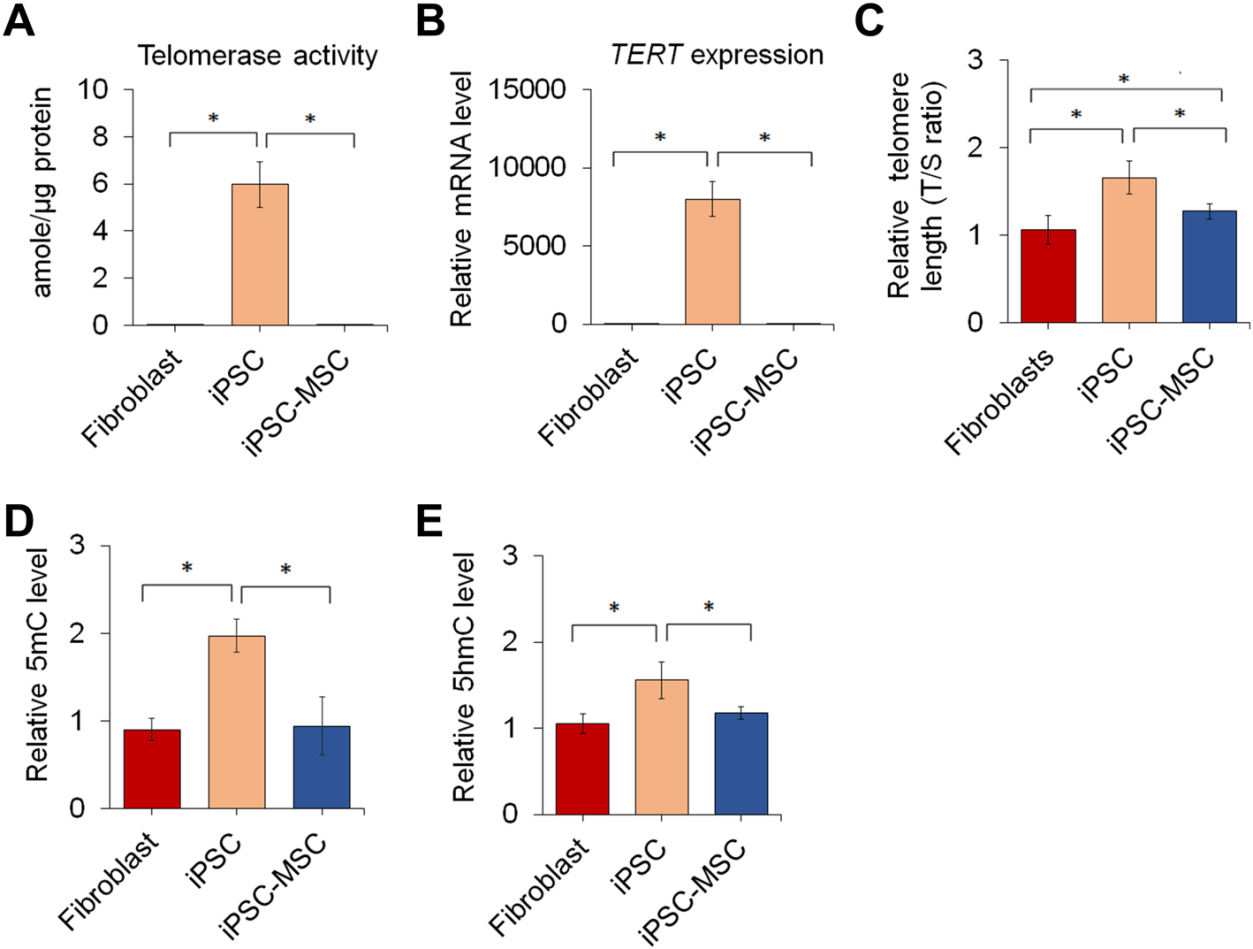
Changes in telomere and DNA methylation of fibroblasts undergoing cellular reprogramming and MSC differentiation. (**A**) Telomerase activity of pig fibroblasts before and after cellular reprogramming and iPSCs after differentiation into MSCs. (**B**) Expression levels of *TERT* in cells at different stages. (**C**) Relative telomere length of cells in response to cellular reprogramming and MSC differentiation induction. T/S ratio: the ratio of the copy number of telomere repeats to that of a single control gene. (**D**) Quantification of global DNA methylation determined by the level of 5mC in different cells. (**E**) Quantification of global DNA hydroxymethylation determined by the level of 5hmC in different cells. *p < 0.05; n = 3.

It has been reported that PSCs exhibit high telomerase activity to maintain long and stable telomeres^25,26^. Yamanaka and others have shown that transcription factor-directed cellular reprogramming results in increased telomerase activity and extended telomere length in iPSCs^27,28^. Hence, we next determined whether the telomere length and telomerase activity of pig cells are affected by cellular reprogramming and MSC differentiation. Our results showed that telomerase activity not detected in fibroblasts was significantly increased after cellular reprogramming, and the increased activity in iPSCs was then diminished and returned to an undetectable level after differentiation into iPSC-MSCs (Fig. 5A). Similarly, telomerase reverse transcriptase (*TERT*) was highly expressed in iPSCs but not in fibroblasts or iPSC-MSCs (Fig. 5B). Furthermore, the telomere length of iPSCs and iPSC-MSCs was significantly greater than that of fibroblasts (Fig. 5C). These results suggest that cellular reprogramming upregulates the expression of *TERT*, which in turn increases telomerase activity to elongate telomeres.

DNA methylation is an epigenetic modification during cell differentiation^29^ or dedifferentiation induced by cellular reprogramming^30^. Previous studies have shown that the level of total DNA methylation in parental cells undergoes a dynamic change during reprogramming^31,32^. To explore the epigenetic characteristics, we analyzed levels of total DNA methylation in our pig cells before and after reprogramming and after MSC differentiation. The results showed that levels of 5-methylcytosine (5mC) and 5-hydroxymethylcytosine (5hmC) were significantly increased after fibroblasts were reprogrammed into iPSCs and then decreased upon MSC differentiation to levels comparable to those of their parental fibroblasts (Fig. 5D,E), indicating that YMS fibroblasts undergo epigenetic changes during cellular reprogramming and MSC differentiation.

### Epigenetic enzyme BAF60A and STAT3 activities correlate with the reprogramming efficiency of miniature pig iPSCs

**Figure 6:**
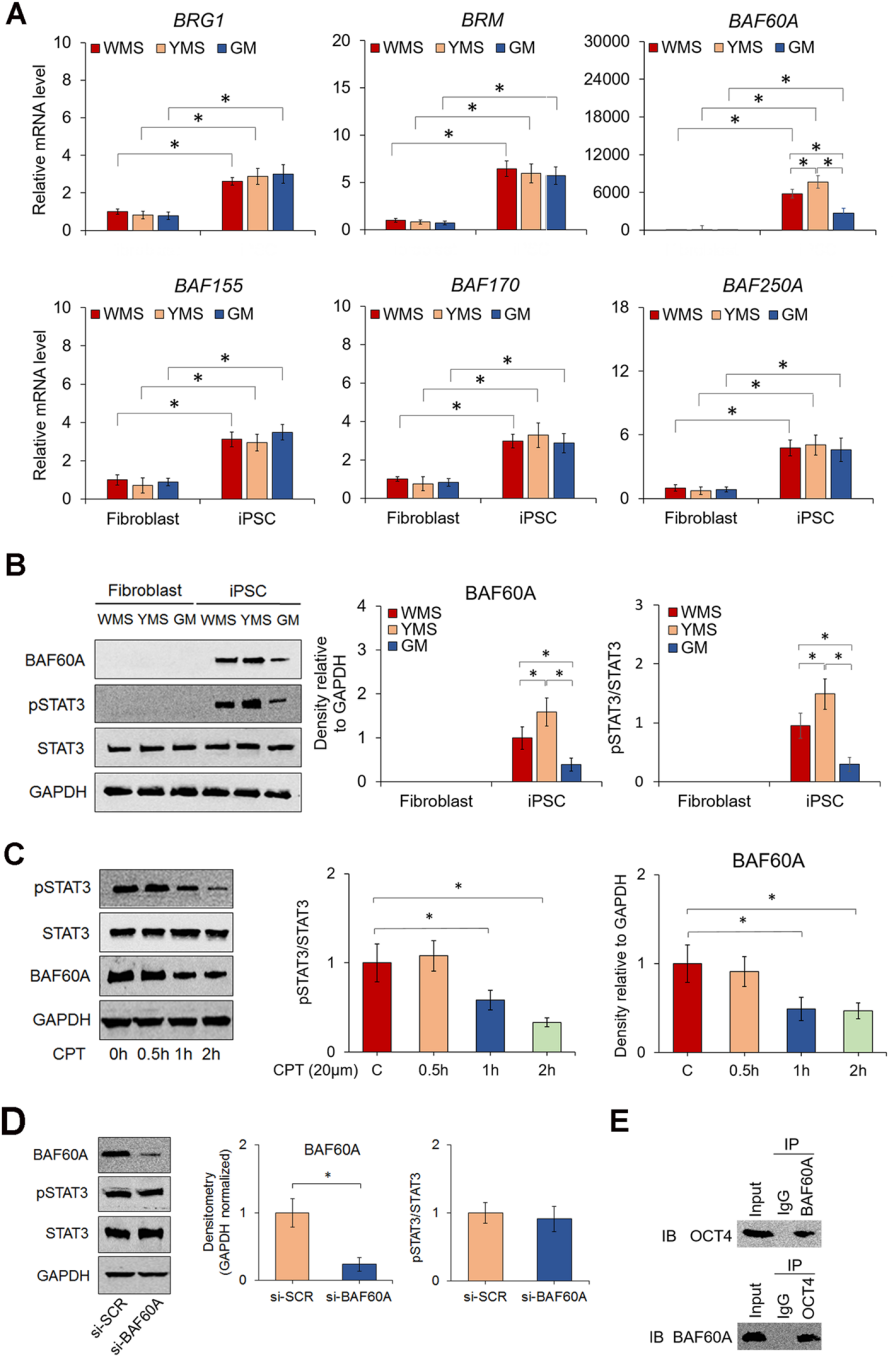
Expression levels of SWI/SNF complexes and STAT3 activity in cells of 3 miniature pig breeds. (**A**) Transcript levels of selected epigenetic enzymes in fibroblasts and iPSCs of different breeds of miniature pigs. (**B**) Images of western blots and quantification of protein bands detecting BAF60A, pSTAT3, and STAT3 expression in cells. GAPDH was used as a loading control. (**C**) Images and quantification of western blots of proteins extracted from YMS iPSCs treated with the STAT3 inhibitor CPT for 2 h. GAPDH was used as a loading control. (**D**) Images and quantification of western blots of proteins extracted from YMS iPSCs treated with BAF60A siRNA or scrambled control. GAPDH was used as a loading control. (**E**) Interaction of BAF60A and OCT4 in YMS iPSC lines analyzed by co-IP. Original western blots are presented in Supplementary Fig. S5. *p < 0.05; n = 3.

It has been reported that the SWI/SNF family plays a critical role in regulating ATP-dependent chromatin remodeling during cellular reprogramming^24^. To determine whether the activities of the chromatin remodeling enzymes are associated with the reprogramming efficiency of cells, we compared the expression of selected SWI/SNF components among parental fibroblasts and iPSCs of the three pig breeds. As shown in Fig. 6A, the mRNA levels of brahma-related gene 1 (*BRG1*), brahma (*BRM*), BRG1-associated factor 60A (*BAF60A)*, *BAF155*, *BAF170*, and *BAF250A* in fibroblasts of each pig breed were significantly increased after cellular reprogramming; however, there was no significant difference in the expression levels of these epigenetic enzymes except *BAF60A* among cells of the different pig breeds. Specifically, YMS iPSCs expressed the highest level of *BAF60A,* and GM iPSCs expressed the lowest level, which correlated positively with the trend of reprogramming efficiency of fibroblasts among the three pig breeds. The transcript expression results were consistent with the protein expression results of BAF60A in both parental fibroblasts and iPSCs (Fig. 6B). As previous studies have shown that LIF/STAT3 signaling is involved in regulating the reprogramming efficiency of cells^33–35^, we examined the activity of STAT3 in cells of pig breeds. The results revealed that STAT3 was increasingly activated in YMS iPSCs compared to WMS or GM iPSCs, consistent with the BAF60A expression levels among cells of the three pig breeds (Fig. 6B). Next, we further analyzed the interaction of BAF60A and STAT3 by treating cells with the STAT3 inhibitor cryptotanshinone (CPT) or *BAF60A* small interfering RNA (siRNA). The results showed that treating iPSCs with 20 µM CPT inhibited STAT3 activation and significantly attenuated the levels of BAF60A in a time-dependent manner (Fig. 6C), but knockdown of *BAF60A* did not affect STAT3 activation (Fig. 6D). These results indicate that the STAT3 pathway regulates the expression of BAF60A. Last, since OCT4 recruits BAFs to target sites to promote the binding of additional pluripotency factors^36,37^, we then determined whether BAF60A binds to OCT4 in YMS iPSCs. The results of coimmunoprecipitation analysis revealed that these two proteins bound together as a complex involved in the activity of cellular reprogramming (Fig. 6E).

### BAF60A regulates colony formation of miniature pig iPSCs

**Figure 7:**
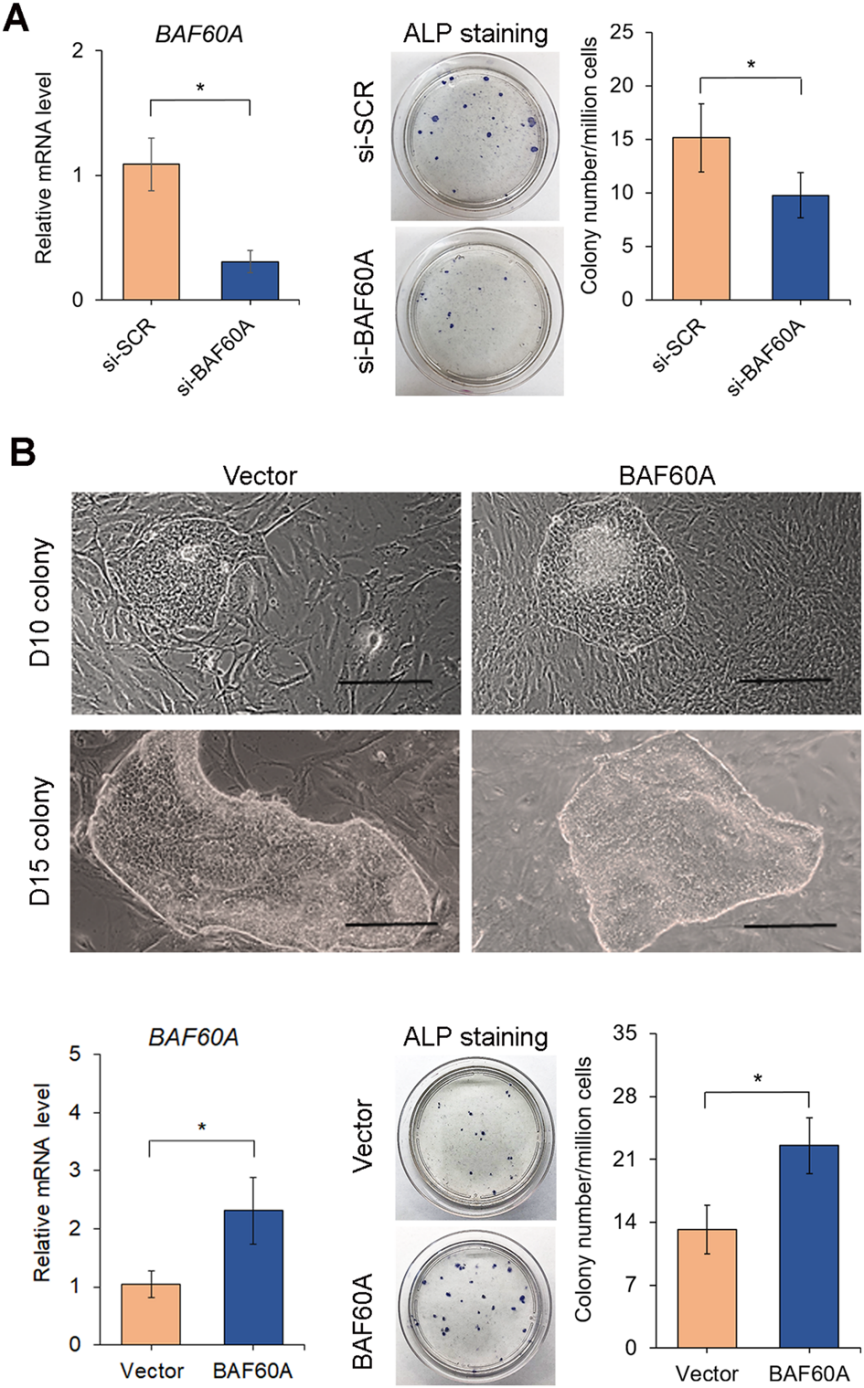
Effects of BAF60A knockdown and overexpression on the reprogramming efficiency of YMS fibroblasts. (**A**) Transcript levels of *BAF60A* in scrambled control and *BAF60A*-knockdown iPSCs (left). Macrographs of iPSC colonies formed in Petri dishes detected by ALP staining 21 days after transfection for *BAF60A* knockdown (middle). Quantification of ALP-positive colonies normalized to total transfected cells (right). (**B**) Colony morphology of control and *BAF60A*-overexpressing iPSCs (top). Transcript levels of *BAF60A* in empty vector control and *BAF60A*-overexpressing iPSCs (bottom left). Macrographs of iPSC colonies detected by ALP staining 21 days after transfection for *BAF60A* overexpression (bottom middle). Quantification of ALP-positive colonies normalized to total transfected cells (bottom right). Scale bar = 200 µm. *p < 0.05; n = 3.

To further determine whether the efficiency of iPSC colony formation shown in Fig. 1D depends on the activity of *BAF60A*, we knocked down *BAF60A* in YMS fibroblasts during cellular reprogramming. The results showed that *BAF60A* knockdown resulted in markedly reduced reprogramming efficiency, as demonstrated by fewer ALP-positive colonies per million transfected cells in a well, compared to the scrambled siRNA control (si-SCR) (Fig. 7A). To confirm the role of *BAF60A*, we transfected YMS fibroblasts with the *BAF60A* plasmid (Addgene, Cat# 21034) to further examine the effect of overexpressing the molecule on iPSC colony formation. As shown in Fig. 7B, *BAF60A* overexpression enhanced the morphology of iPSC colonies with clearly defined borders. In addition, significantly more colonies formed in the *BAF60A*-overexpressing culture, indicating greater reprogramming efficiency than in the control culture. Similar to the result of YMS fibroblasts, the reprogramming efficiency of WMS and GM fibroblasts was significantly enhanced with the overexpression of *BAF60A* (Supplementary Fig. S2), indicating that *BAF60A* is a critical chromatin remodeling enzyme that controls the efficiency of iPSC formation across different breeds of miniature pigs.

## Discussion

While success in reprogramming porcine cells into iPSCs by overexpressing pluripotency factors has been reported^8–11^, it remains challenging to efficiently generate porcine iPSCs through nonviral reprogramming approaches. Here, we have demonstrated that fibroblasts of three different breeds of miniature pigs are successfully reprogrammed into iPSCs using the episomal vector pMaster 12, and cellular reprogramming of YMS fibroblasts is significantly more efficient than that of WMS and GM fibroblasts. In addition, we identified STAT3 signaling and the epigenetic enzyme BAF60A involved in the regulation of iPSC colony formation and demonstrated that the differential activities of these molecules correlate with the reprogramming efficiencies of fibroblasts among the three breeds of miniature pigs. Importantly, we have also shown that increasing the BAF60A activity of pig fibroblasts can promote the formation of iPSC colonies.

The reprogramming efficiency of pig fibroblasts acquired in the current study ranged from 0.0006% of wild-type GM cells to 0.0022% of *BAF60A*-overexpressing YMS cells. Compared to the data reported in the previous study using the episomal approach, our results of BAF60A overexpression show an enhancement in reprogramming efficiency; however, there is still a gap between the efficiencies that we report here and those shown in previous studies using the lentiviral approach (Supplementary Table S1). Although more efficient than nonviral approaches to generate iPSCs, virus-based reprogramming methods are unfeasible for clinical applications because of possible insertional mutations of DNA that may influence differentiation potential or even result in tumorigenesis^38^. In contrast, nonviral reprogramming approaches such as episomal vector-based transfection can generate transgene-free iPSCs to avoid safety concerns associated with potential insertional mutagenesis and residual expression of transgenes^39^.

Much to our surprise, the YMS iPSC lines generated in this study using the episomal vector reprogramming method were not free of transgenes. Except for *NANOG* and *LIN28*, the four exogenous Yamanaka factors remained in the established iPSC lines. A similar finding was also shown in a previously reported study, in which exogenous Yamanaka factors remained in pig iPSCs over 30 passages^17^, presenting an unresolved challenge. It is unclear why the episomal vector approach commonly used to generate transgene-free human and mouse iPSCs^39–41^ fails to do the same with pig cells. Future studies may focus on enhancing the episomal vector and optimizing the reprogramming protocol and culture medium to improve the generation of transgene-free pig iPSCs.

We show that the use of our iPSC medium and a feeder layer of mouse embryo fibroblasts (MEFs) can maintain undifferentiated colonies of miniature pig iPSCs in culture for an extensive period of time (Supplementary Fig. S3). The composition of our iPSC medium is different from those previously reported for pig iPSC culture. In efforts to create our iPSC culture medium, we first used 2i/LIF medium^17^ to maintain YMS iPSCs, but the cells died after being passaged. We then switched to Essential 8 (E8) medium, a commonly used human iPSC medium^42,43^, and found that it kept YMS iPSCs alive but failed to prevent the cell from undergoing spontaneous differentiation. Given that LIF/STAT3 signaling plays a critical role in supporting the pluripotency of pig iPSCs^12,44,45^, we added LIF to the E8 medium. In addition, with a previous study showing that supplementation with activin A and WNT signaling regulators, CHIR99021 and IWR-1, helps maintain the pluripotency of pig ESCs while refraining spontaneous differentiation of the cell^46^, we added these molecules to the E8/LIF medium to finalize the composition of our pig iPSC culture medium. Furthermore, we used MEFs as a feeder layer to support the long-term growth of undifferentiated pig iPSCs in culture^47^*.* In our initial work, we tried to adopt feeder-free culture and maintained pig iPSCs on Matrigel matrix but found that the iPSCs underwent spontaneous differentiation in such a culture setup. Similar to most of the previously reported studies using MEFs in iPSC culture^10,16,17^, our current work demonstrates the necessity of including MEFs in culture to maintain miniature pig iPSCs before further optimization to eliminate them for feeder-free culture.

In the current study, we demonstrate that the efficiency of iPSC generation from fibroblasts of different pig breeds is positively correlated with the level of BAF60A in reprogrammed cells. Using loss- and gain-of-function assays, we determined that BAF60A is a crucial molecule regulating iPSC colony formation during cellular reprogramming. Previous studies have shown that components of the BAF complex, BRG1 and BAF155, are key proteins facilitating Yamanaka factor binding to target promoters during cellular reprogramming^23,48^. Here, we demonstrate that in addition to *BRG1* and *BAF155*, other family members, *BRM*, *BAF60A*, *BAF170*, and *BAF 250A,* are increasingly expressed in iPSCs, indicating that these components may be involved in the epigenetic regulation of cellular reprogramming. Our results further reveal the role of BAF60A in controlling the reprogramming efficiency of fibroblasts of different pig breeds, similar to a previously reported finding that BAF60A and other members of the SWI/SNF family are critical in regulating iPSC generation of donors with different ancestry^24^. These findings provide a possible explanation of the biological cause leading to differences in the efficiency of iPSC generation between different ethnic groups or strains of the same species.

The potential of pig iPSC derivatives for cell therapies is explored in this study. Particularly, MSCs derived from iPSCs are considered cells of interest for musculoskeletal regeneration applications owing to the advantages of unlimited cell supply^49,50^ and the capability of renewal and multilineage differentiation^51–54^ over adult tissue-derived counterparts such as bone marrow MSCs. In this study, using a large animal model, we reprogrammed YMS fibroblasts into iPSCs and then differentiated them into MSCs to demonstrate their potential applications for tissue regeneration. Our results show that pig iPSC-MSC lines express phenotypic characteristics of MSCs and can become bone, cartilage, and fat cells. Notably, unlike their parental iPSCs, derived iPSC-MSCs are free of exogenous reprogramming factors and do not lead to teratoma formation, implying the safety of iPSC-MSCs for therapeutic applications.

The analysis of telomere length shows that iPSC-MSCs have longer telomeres than their parental fibroblasts, indicating that through the process of cellular reprogramming, cells can increase *TERT* expression and telomerase activity to restore telomere length that becomes shorter with each cell division. As shown in our previous and others’ studies^53–55^, iPSC-MSCs with extended telomeres exhibit characteristics and activities of youthful MSCs and can undergo more population doublings. In addition, we found that cellular reprogramming alters the DNA methylation of pig cells, consistent with previously reported finding^31,32^. For example, a genome-wide analysis has shown that DNA regions of iPSCs are differentially methylated compared with those of parental fibroblasts^56^. In this study, we demonstrate that the level of global DNA methylation undergoes a dynamic change, increasing during cellular reprogramming and then decreasing upon MSC differentiation. These results provide insight into changes in DNA, including telomeres, induced by cellular reprogramming and lineage-specific differentiation in miniature pig cells.

Several previous studies have demonstrated the generation of pig iPSCs by Yamanaka factor-mediated cellular reprogramming. Compared to the methods used in these studies, ours has an advantage in generating potentially safer iPSCs for applications over virus-directed approaches^10,15,16^ and another advantage in enhancing the reprogramming efficiency of pig cells over the same episomal vector-directed approach^17^. However, the presence of an MEF feeder layer introducing an exogenous factor in pig iPSC culture is considered a limitation of our method. Further optimization of iPSC culture by replacing MEFs with a chemically defined matrix substrate to overcome this limitation is underway in our laboratory.

Taken together, we have demonstrated that using the episomal plasmid pMaster12 and our culture medium, fibroblasts harvested from three breeds of miniature pigs are successfully reprogrammed into iPSCs. Among the different pig breeds, the reprogramming efficiency of YMS fibroblasts is higher than that of WMS or GM fibroblasts, which is dependent on the expression level of BAF60A, a subunit of the SWI/SNF chromatin-remodeling complexes. We also found that the expression of BAF60A is regulated by STAT3 signaling. To the best of our knowledge, a similar study comparing the efficiency of iPSC generation between different pig breeds has not been reported. Our findings also provide insight into a molecular mechanism governing the efficiency of cellular reprogramming for the generation of miniature pig iPSCs.

## Materials and methods

### Fibroblast isolation and culture

All animal experiments and procedures reported here are in accordance with Animal Research: Reporting of In Vivo Experiments guidelines 2.0. The animal protocol (ID # V005016-R02) was approved by the Institutional Animal Care and Use Committee (IACUC) of the University of Wisconsin-Madison, and all methods to harvest animal tissue were carried out in accordance with relevant guidelines and regulations. Fibroblasts were isolated from ear notches of three breeds of miniature pigs, YMS, GM, and WMS (details about the animals are provided in Supplementary Table S2). Both male and female animals between newborn and 4 days of age were used for fibroblast isolation. Briefly, collected dermal tissue was incubated with digestion medium of collagenase/dispase for 2 h before adding an equal amount of complete medium composed of low-glucose Dulbecco Modified Eagle Medium (DMEM), 10% fetal bovine serum (FBS; Atlanta Biologicals, Atlanta, GA, USA), and antibiotics to stop digestion. The solution was then filtered through a 70-µm cell strainer and centrifuged at 300 × *g* for 5 min. Cells were resuspended and plated in culture flasks with complete medium and maintained in an incubator at 37 °C in a humidified 5% CO_2_ atmosphere.

### Generation of iPSCs and determination of reprogramming efficiency

For the generation of miniature pig iPSCs, 10^6^ fibroblasts at P3 were transfected with 4 μg of episomal plasmid pMaster12 (Addgene # 58527)^17^ using Nucleofector™ II with the A-024 program (Amaxa, Walkersville, MD, USA) and the fibroblast-specific Nucleofector kit (Lonza, Hayward, CA, USA). Transfected cells were plated on a feeder layer of γ-ray-treated MEFs in a 6-well plate containing modified E8 medium (E8 medium supplemented with 5 ng/ml activin A, 1.5 µM CHIR99021, 2.5 µM IWR-1, and 10 ng/ml LIF). The medium was replaced with fresh modified E8 medium and G418 (400 µg/ml) the next day and then maintained for 5 days before switching back to modified E8 medium. After 3 weeks, cell colonies were picked under a microscope. Each colony was individually transferred to a well of 24-well plates containing MEF feeder layers with modified E8 medium for expansion and passaging afterward. The SIGMAFAST BCIP/NBT kit (Sigma–Aldrich, Burlington, MA, USA) was used to stain iPSCs for ALP activity following the manufacturer’s instructions. The reprogramming efficiency of fibroblasts was determined by counting the number of iPSC colonies positive for ALP staining per million cells in culture 21 days after transfection.

### Teratoma formation assay and karyotyping

The animal protocol (ID # M005566-R01) to perform the teratoma assay was approved by the IACUC of the University of Wisconsin-Madison, and all methods were carried out in accordance with relevant guidelines and regulations. Briefly, 10^6^ iPSCs were resuspended in 100 µL of 50% Matrigel in DMEM and Ham’s F-12 Nutrient Mixture and injected subcutaneously into the hind leg of NOD. Cg-Prkdc^scid^Il2rg^tm1wjl^/SzJ mice (The Jackson Laboratory, Bar Harbor, ME, USA). Eight weeks after injection, teratomas were harvested, dissected, and fixed with 4% paraformaldehyde. Paraffin-embedded tissue was sliced and stained with H&E. Standard G-banded karyotyping was carried out and interpreted by Cell Line Genetics (Madison, WI, USA).

### Assessment of pluripotency of iPSCs into three germ layer cells

Established iPSCs were induced to differentiate into mesodermal, endodermal, and ectodermal lineage cells in culture using the STEMdiff™ Trilineage Differentiation kit (STEMCELL Technologies). Quantitative RT–PCR detecting the expression of *TBXT*/*CXCR4*, *SOX17/FOXA2, and PAX6/NES* was performed to identify mesodermal, endodermal, and ectodermal cells, respectively.

### Derivation of MSCs from iPSCs

Three individual YMS iPSC lines were induced by the STEMdiff™ Mesenchymal Progenitor kit (STEMCELL Technologies, Vancouver, BC, Canada) to generate iPSC-MSCs following the manufacturer’s instructions. Briefly, iPSCs were cultured in modified E8 medium until reaching 80% confluence and then induced by STEMdiff™-ACF Mesenchymal Induction Medium for 4 days with daily medium change, followed by complete MesenCult™-ACF Plus Medium for another 2 days with daily medium change. Cells were then passaged as P1 iPSC-MSCs and cultured in complete MesenCult™-ACF Plus Medium for another 4 passages. The culture medium was then switched to basal growth medium composed of DMEM, 10% FBS, and antibiotics to maintain iPSC-MSCs.

### Determination of multilineage differentiation of iPSC-MSCs

Adipogenic, chondrogenic, and osteogenic differentiation of iPSC-MSCs was induced as previously described^57^. Briefly, cells were cultured in adipogenic, chondrogenic, or osteogenic medium for differentiation. After 21 days of induction, osteogenic differentiation was determined by Alizarin red S staining (Rowley Biochemical, Danvers, MA, USA) and calcium quantification. Adipogenic differentiation was analyzed by Oil red O staining (Sigma–Aldrich, St. Louis, MO, USA). Chondrogenic differentiation was assessed by Alcian blue staining and GAG quantification. In addition to histological and biochemical analyses, the transcript expression of bone-associated (*CBFA1, OC, ALP*), cartilage-associated (*SOX9, COL2, ACAN*)*,* and fat-associated (*PPARG2, LPL*) markers was analyzed by quantitative RT–PCR.

### Knockdown and overexpression of *BAF60A*

Synthetic siRNA designed to silence *BAF60A* and control scrambled siRNA were purchased from Integrated DNA Technologies (Coralville, Iowa, USA). The sequences of the *BAF60A* siRNA are GUGGUAAUCAGUGCAUUGAAUGGAC for the sense strand and GUCCAUUCAAUGCACUGAUUACCACUA for the antisense strand. Cells were transfected with *BAF60A* or scrambled siRNA using GenMute siRNA transfection reagent (SignaGen Laboratories, Gaithersburg, MD) at day 6 of cellular reprogramming. To overexpress *BAF60A, the pBS-hBAF60A* plasmid (Addgene, Cat #21034) or *pBlueScript SK(-)* empty vector (Agilent, Cat #2102206) was electroporated into cells at the beginning of cellular reprogramming.

### Total RNA extraction and quantitative RT–PCR

Total RNA was extracted from cells using the Zymo Quick-RNA MicroPrep kit (Zymo Research, Irvine, CA, USA). To synthesize cDNA, one microgram of total RNA was used in each reaction with the High-Capacity cDNA Reverse Transcription kit (Applied Biosystems, Carlsbad, CA, USA). Quantitative RT–PCR was performed using iQ SYBR Green Premix (Bio–Rad, Hercules, CA, USA) with primers detecting *CBFA1, ALP, OC, SOX9*, *COL2*, *ACAN, PPARG2*, *LPL, TERT*, *OCT4*, *SOX2*, *NANOG*, *TBXT, CXCR4*, *PAX6*, *NES*, *SOX17*, *FOXA2*, *BRG1*, *BRM*, *BAF60A*, *BAF155*, *BAF170*, *BAF250A,* and ubiquitin C (*UBC*). Sequences of the primers obtained from previous resports^17,58^ or designed using NCBI Primer Blast are listed in Supplementary Table S3. The 2^−ΔCT^ method was used to determine the relative expression level of a target transcript to that of *UBC* as an internal control.

### PCR and electrophoresis

To detect genomic integration of transgenes in miniature pig iPSCs and their derivatives, total RNA and cDNA were prepared as described above. PCRs were carried out with 2X PCR Master Mix (Promega, Madison, WI, USA) using the following program: initial denaturation at 94 °C for 5 min; 32 cycles of annealing at 94 °C for 15 s, 55 °C for 30 s, and 68 °C for 1 min; and extension at 68 °C for 7 min. PCR products were separated by electrophoresis on a 1% agarose gel containing 0.5 µg/ml ethidium bromide and visualized by UV illumination.

### Protein extraction and western blotting analysis

Cells were lysed in RIPA buffer and then centrifuged at 14,000×*g* for 10 min to collect the supernatant. The protein concentration in the supernatant was measured using the BCA Protein Assay kit (Pierce, Rockford, IL, USA). A 20-μg protein sample was loaded into each lane of a 10% polyacrylamide gel (Bio–Rad, Hercules, CA, USA) for electrophoresis. Separated proteins were then transferred from the gel onto a nitrocellulose membrane (Bio–Rad). The membrane was incubated with primary antibodies against BAF60A, pSTAT3, and STAT3 (Thermo Fisher Scientific, Waltham, MA, USA) in a blocking solution composed of Tris-buffered saline containing 5% nonfat milk (Bio–Rad) and 0.1% Tween 20 (Sigma–Aldrich) overnight at 4 °C. After removing unbound antibodies, the membrane was incubated with horseradish peroxidase-linked secondary antibody (Cell Signaling Technology, Danvers, MA, USA) in the blocking solution for 1 h at room temperature. Immuno-detected protein bands on the membrane were visualized using SuperSignal West Pico Chemiluminescent Substrate (Pierce) and then documented by the Kodak Image Station 4000R Pro system (Kodak, Rochester, NY, USA). Information on the antibodies used in this study is provided in Supplementary Table S4.

### Coimmunoprecipitation analysis (co-IP)

Cell extracts were prepared using lysis buffer as described above. Lysates were incubated overnight at 4 °C with antibodies against OCT4 (Novus Biologicals, Littleton, CO, USA), BAF60A, or IgG control (Thermo Fisher Scientific). Antibody-antigen complexes were then incubated with Protein A/G PLUS-Agarose (Santa Cruz Biotechnology, Dallas, Texas, USA) for 2 h before washing with lysis buffer 3 times, resuspending in SDS gel loading buffer, and boiling for 5 min. The boiled samples were loaded on a 10% SDS–polyacrylamide gel for western blotting analysis.

### Immunofluorescence analysis

Cells grown in glass-bottom dishes (MatTek, Ashland, MA, USA) were fixed with 4% paraformaldehyde, permeabilized and blocked with 0.3% Triton X-100 and 1% BSA in PBS and incubated overnight at 4 °C with primary antibodies specific for NANOG, OCT4, or SOX2 (R&D Systems, Minneapolis, MN, USA). Donkey anti-goat IgG NL493 or donkey anti-mouse IgG Alexa Fluor 546 (Thermo Fisher Scientific) at 1:200 was used as the secondary antibody to treat the cells. Staining of 4,6-diamidino-2-phenylindole and dihydrochloride (DAPI) was performed to visualize nuclei before imaging by a laser scanning confocal microscope (Nikon A1RS, Japan).

### Flow cytometry analysis

Cells were trypsinized and washed twice using flow cytometry staining buffer made of ice-cold PBS, 0.1% sodium azide, and 1% bovine serum albumin (Sigma–Aldrich). Antibodies detecting the surface markers CD90, CD29, CD44, CD45, and CD34 to identify MSCs were used together with or without secondary antibodies for analysis. Detailed information on the antibodies used in this study is provided in Supplementary Table S4. The expression of the surface markers was analyzed by MACSQuant Analyzer 10 (Miltenyi Biotec, Bergisch Gladbach, Germany). Data were analyzed using FlowJo software (TreeStar, Ashland, OR, USA).

### Telomerase activity measurement

Telomerase activity was determined by the TRAPeze^®^ RT Telomerase Detection kit (Millipore, Billerica, MA, USA) following the manufacturer’s instructions. Cell extracts were prepared using the CHAPS lysis buffer provided in the kit, and protein concentrations were measured using the BCA Protein Assay kit (Pierce). One microgram of total protein from each sample was added to each reaction. Heat-treated controls of each sample were included to rule out false-positive signals from PCR artifacts.

### Determination of relative telomere length

Relative telomere length was determined by the method described by Cawthon et al.^59^ with minor modifications. Briefly, DNA was extracted from cells using the Zymo Quick-DNA MicroPrep kit following the manufacturer’s instructions. Telomere length was quantified using quantitative RT–PCR by comparing the telomere repeat sequence to a single copy gene (*36B4*). The reaction mixture consisted of iQ SYBR Green Premix (Bio–Rad), forward and reverse primers and 35 ng of DNA per reaction. Serial dilutions of reference samples for telomeres and *36B4* PCRs were included to generate a standard curve. A standard curve plot showing Ct versus logarithm of the amount of input reference DNA was constructed based on the quantitative RT–PCR results. The telomere repeat copy number (T) and single control gene copy number (S) in each sample were determined by comparison to the standard curve. The relative telomere length of each sample was measured by calculating the T/S ratio.

### Quantification of DNA methylation and hydroxymethylation

Genomic DNA was extracted from cells using the Zymo Quick-DNA MicroPrep kit following the manufacturer's instructions. Methylation and hydroxymethylation levels of genomic DNA were determined by MethylFlash Global DNA methylation (5mC) and hydroxymethylation (5hmC) ELISA Easy kits (Epigentek, Farmingdale, NY, USA) following the manufacturer's protocols.

### Statistical analysis

All quantitative data were collected from assays with three biological replicates (n = 3) for each group and presented as the mean ± standard deviation. Student’s t test or one-way analysis of variance (ANOVA) with post hoc Tukey’s test was used for statistical comparison. A p value less than 0.05 was considered statistically significant.

## Supplementary Information

Supplementary Figures.

Supplementary Table S1.

Supplementary Table S2.

Supplementary Table S3.

Supplementary Table S4.
