# Author Correction: Epigenetic regulation of BAF60A determines efficiency of miniature swine iPSC generation

**DOI:** 10.1038/s41598-022-14878-4

**Published:** 2022-06-17

**Authors:** Hongli Jiao, Ming‑Song Lee, Athillesh Sivapatham, Ellen M. Leiferman, Wan‑Ju Li

**Affiliations:** 1 Laboratory of Musculoskeletal Biology and Regenerative Medicine, Department of Orthopedics and Rehabilitation, University of Wisconsin-Madison, 1111 Highland Ave, WIMR 5051, Madison, WI 53705 USA; 2 Department of Biomedical Engineering, University of Wisconsin-Madison, Madison, WI USA

Correction to: *Scientific Reports* 10.1038/s41598-022-12919-6, published online 31 May 2022

The original version of this Article contained an error in the Materials and methods, under the subheading ‘Generation of iPSCs and determination of reprogramming efficiency’,

“For the generation of miniature pig iPSCs, 106 fibroblasts at P3 were transfected with 4 μg of episomal plasmid pMaster12 (Addgene # 58527)^17^ using Nucleofector™ II with the A-024 program (Amaxa, Walkersville, MD, USA) and the fibroblast-specific Nucleofector kit (Lonza, Hayward, CA, USA).”

now reads:

“For the generation of miniature pig iPSCs, 10^6^ fibroblasts at P3 were transfected with 4 μg of episomal plasmid pMaster12 (Addgene # 58527)^17^ using Nucleofector™ II with the A-024 program (Amaxa, Walkersville, MD, USA) and the fibroblast-specific Nucleofector kit (Lonza, Hayward, CA, USA).”

The original Article has been corrected.

