# Supplementary material for: Epigenetic regulation of BAF60A determines efficiency of miniature swine iPSC generation
Source: Sci Rep. 2022 May 31;12:9039. doi: 10.1038/s41598-022-12919-6 (PMC9156668; doi:10.1038/s41598-022-12919-6)
Supplement: Supplementary file 1 — Supplementary Figures. [file 41598_2022_12919_MOESM1_ESM.docx]

**Supplementary Figures**

**
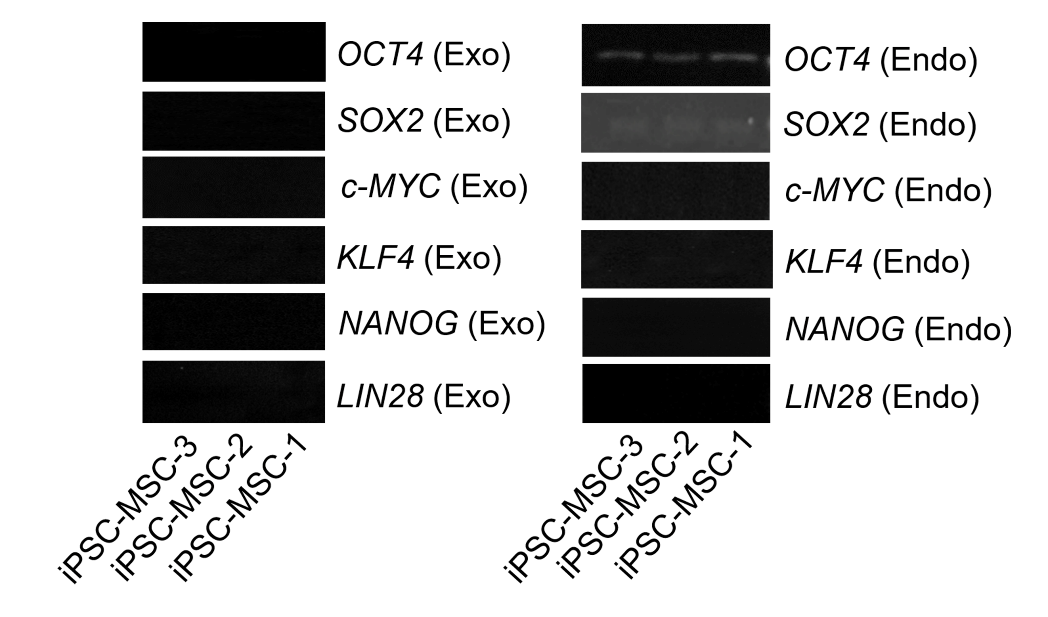
**

**Supplementary Figure S1**. Transcript expression of exogenous and endogenous pluripotency markers in 3 YMS iPSC-MSC lines.

**
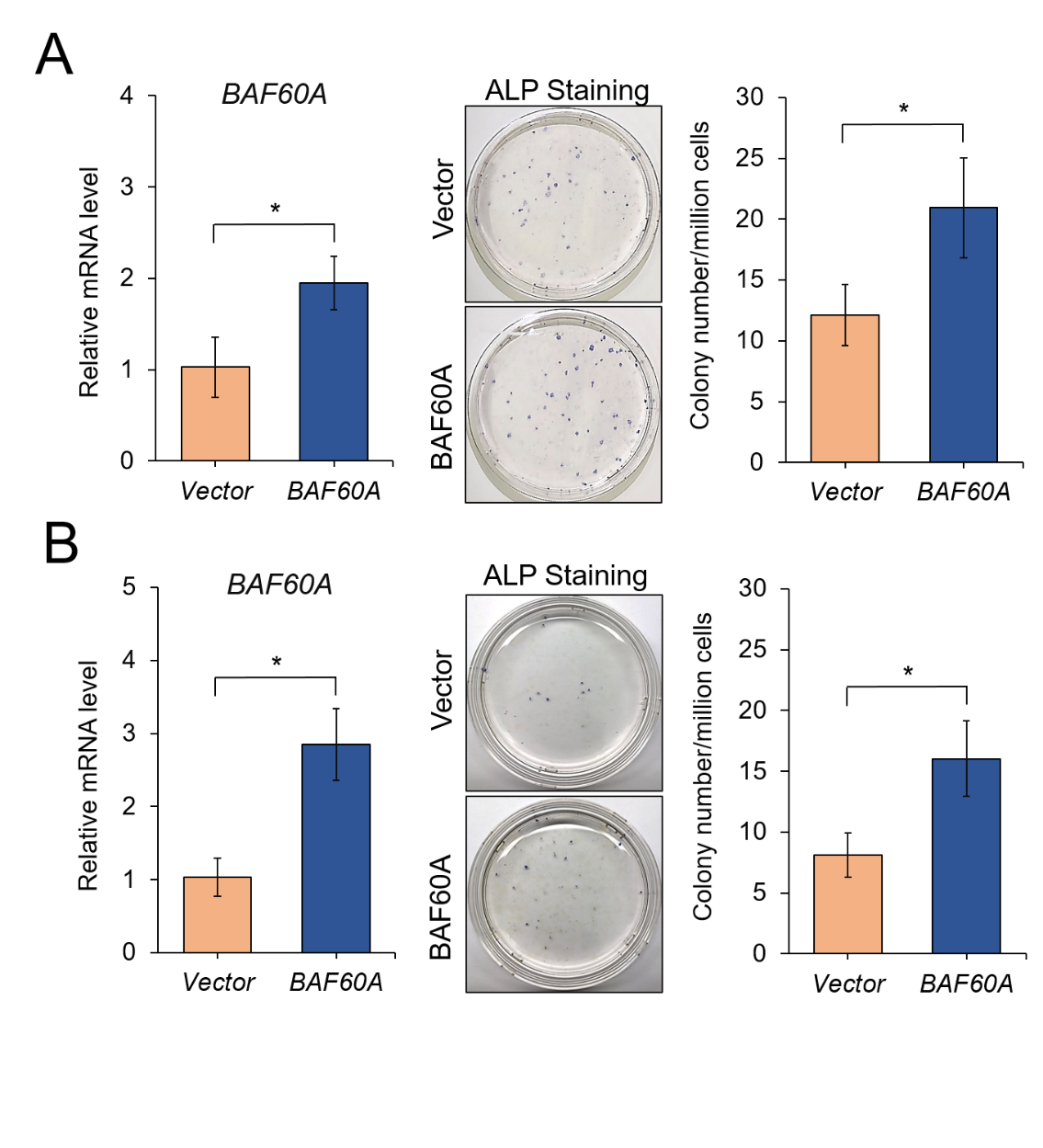
**

**Supplementary Figure S2**. Effects of BAF60A overexpression on the reprogramming efficiency of fibroblasts from WMS (A) or GM (B). Transcript levels of *BAF60A* in empty vector control and *BAF60A*-overexpressing iPSCs (left). Macrographs of iPSC colonies detected by ALP staining 21 days after transfection for *BAF60A* overexpression (middle). Quantification of ALP-positive colonies normalized to total transfected cells (right). **p* < 0.05; n = 3.

**
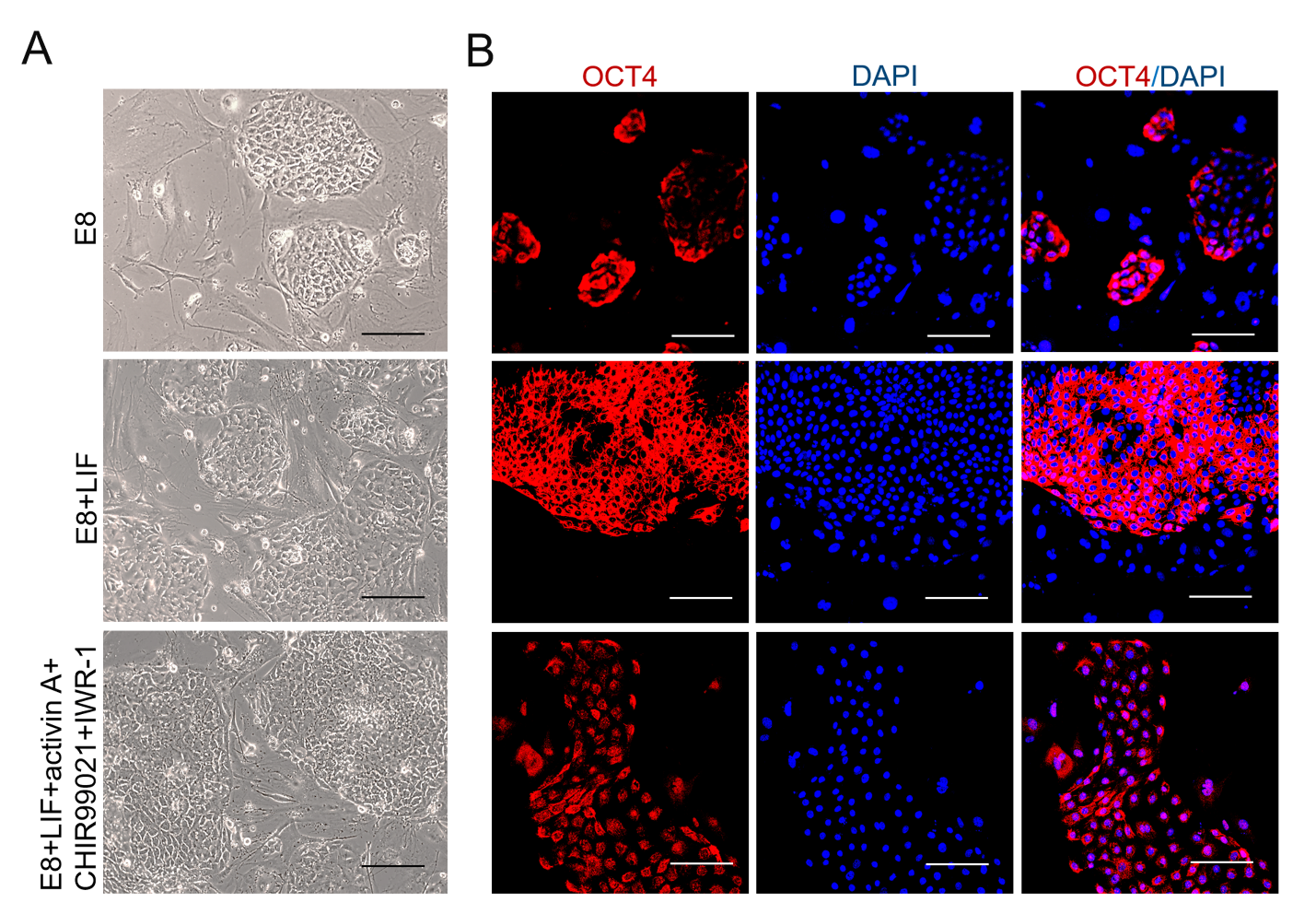
**

**Supplementary Figure S3**. Colony formation of miniature pig iPSCs cultured with different compositions of medium. (A) Morphology of iPSC colonies derived from YMS fibroblasts analyzed by bright field images. Scale bar = 200 µm. (B) Immunofluorescent images of OCT4-positive iPSC colonies derived from YMS fibroblasts. Scale bar = 100 µm.


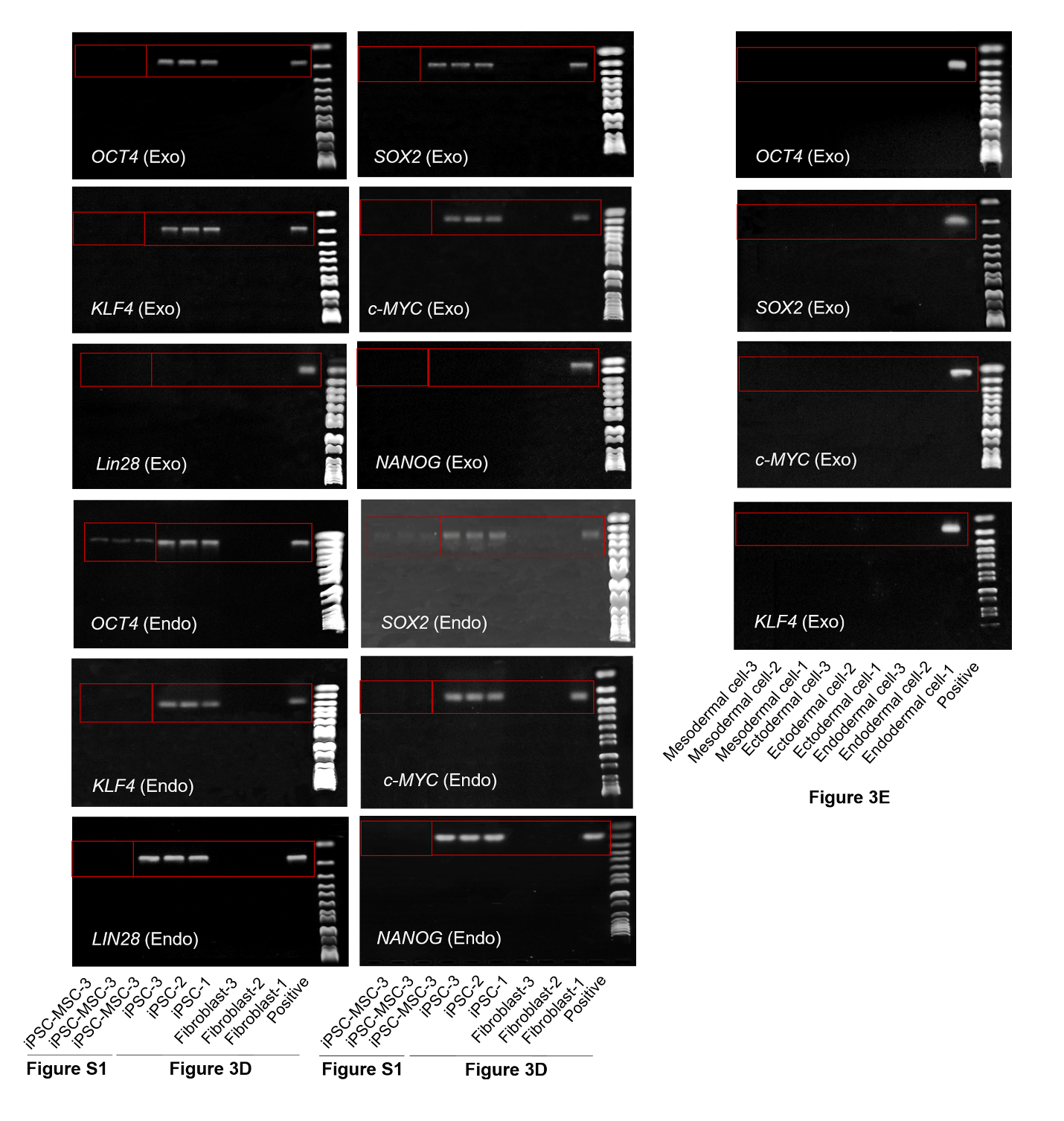


**Supplementary Figure S4**. Original images of agarose gel electrophoresis in Figure 3 and Supplementary Figure S1.

**
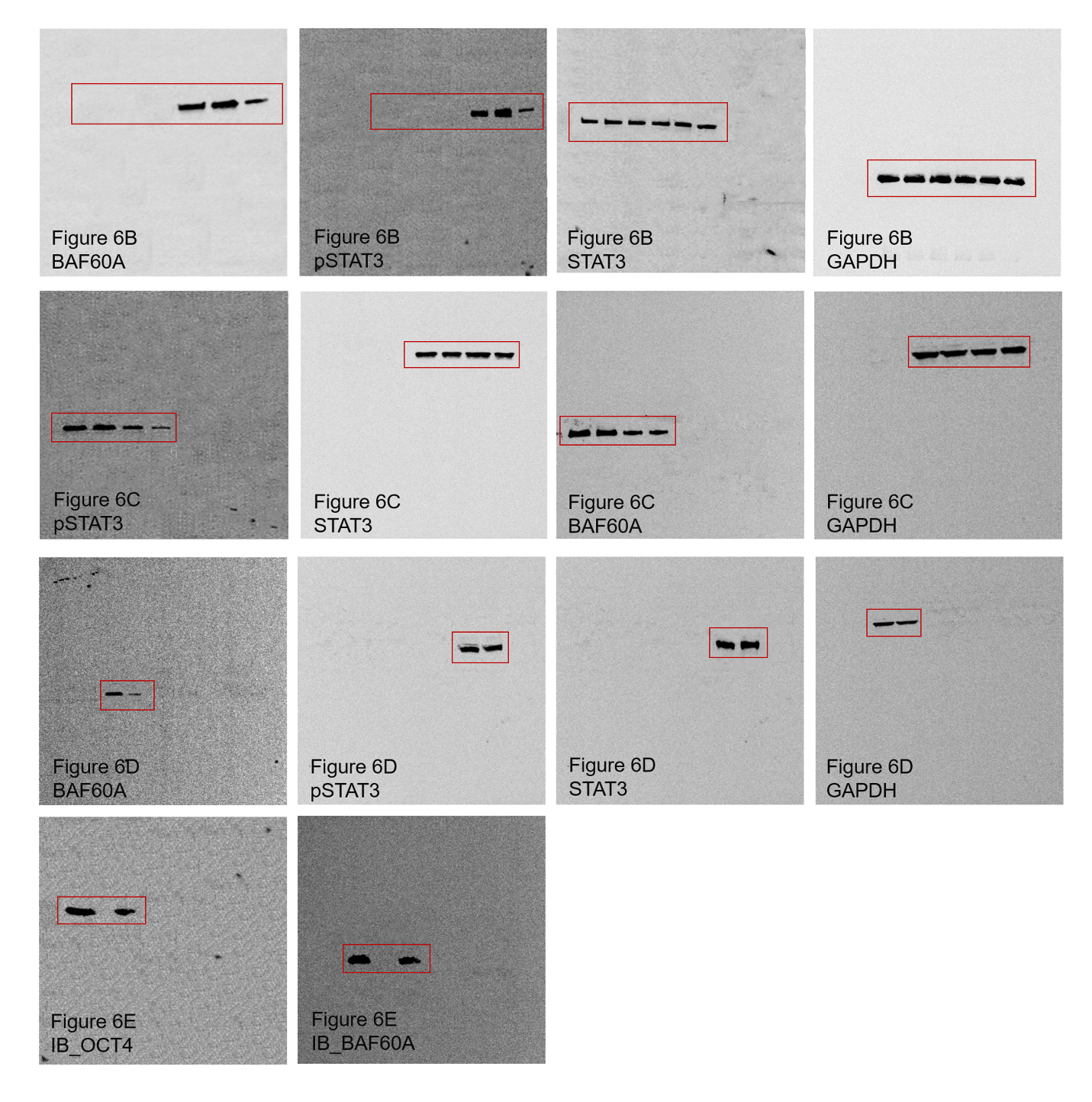
**

**Supplementary Figure S5**. Original images of western blots in Figure 6.
