# Supplementary material for: Epigenetic regulation of BAF60A determines efficiency of miniature swine iPSC generation
Source: Sci Rep. 2022 May 31;12:9039. doi: 10.1038/s41598-022-12919-6 (PMC9156668; doi:10.1038/s41598-022-12919-6)
Supplement: Supplementary file 2 — Supplementary Table S1. [file 41598_2022_12919_MOESM2_ESM.docx]

**Table S1 Summary of iPSC Generation from Miniature Pig Fibroblasts Reported in Previous and Current Studies**

| **Publication** | **Reprogramming Method** | **Cell Type** | **BAF60A Expression Manipulation** | **Reprogramming Efficiency** |
| --- | --- | --- | --- | --- |
| Ezashi et al., 2009 [10] | Lentiviral | Porcine fetal fibroblasts | None | 0.1% |
| Chen et al., 2021 [15] | Lentiviral | Porcine fetal fibroblasts | None | 0.23% |
| Fukuda et al., 2017 [16] | Lentiviral | Porcine fetal fibroblasts | None | 0.05% |
| Du et al., 2015  [17] | Episomal | Porcine fetal fibroblasts | None | 0.001% |
|  | | | | |
| Our current report | Episomal | YMS ear fibroblasts | None | 0.0015% |
|  |  |  | BAF60A knockdown | 0.0009% |
|  |  |  | BAF60A overexpression | 0.0022% |
| Our current report | Episomal | WMS ear fibroblasts | None | 0.0011% |
|  |  |  | BAF60A overexpression | 0.0020% |
| Our current report | Episomal | GM ear fibroblasts | None | 0.0006% |
|  |  |  | BAF60A overexpression | 0.0016% |
