# Supplementary material for: Epigenetic regulation of BAF60A determines efficiency of miniature swine iPSC generation
Source: Sci Rep. 2022 May 31;12:9039. doi: 10.1038/s41598-022-12919-6 (PMC9156668; doi:10.1038/s41598-022-12919-6)
Supplement: Supplementary file 3 — Supplementary Table S2. [file 41598_2022_12919_MOESM3_ESM.docx]

**Table S2 Information of Three Breeds of Miniature Pigs**

| **Breed** | **ID** | **DOB** | **Date of Ear Notch Collection** | **Sex** |
| --- | --- | --- | --- | --- |
| WMS | 19-4491-1 | 5/10/2019 | 5/14/2019 | Gilt |
|  | 19-4496-2 | 5/10/2019 | 5/14/2019 | Boar |
|  | 19-4471-1 | 5/12/2019 | 5/14/2019 | Gilt |
| GM | 3051668 | 5/22/2019 | 5/24/2019 | Gilt |
|  | 3051641 | 5/22/2019 | 5/24/2019 | Boar |
|  | 3051692 | 5/22/2019 | 5/24/2019 | Gilt |
| YMS | 9763F | 8/19/2019 | 8/19/2019 | Gilt |
|  | 9763M | 8/19/2019 | 8/19/2019 | Boar |
|  | 9432F | 8/19/2019 | 8/19/2019 | Gilt |
