# Supplementary material for: Epigenetic regulation of BAF60A determines efficiency of miniature swine iPSC generation
Source: Sci Rep. 2022 May 31;12:9039. doi: 10.1038/s41598-022-12919-6 (PMC9156668; doi:10.1038/s41598-022-12919-6)
Supplement: Supplementary file 4 — Supplementary Table S3. [file 41598_2022_12919_MOESM4_ESM.docx]

Table S3 **Primer sequences for quantitative RT-PCR**

| **Genes** | **Forward primer sequence** | **Reverse primer sequence** |
| --- | --- | --- |
| **Porcine** | | |
| *SOX9* | GACAGGAACCTGGACTTGAAAC | GTCCAAACAGGCAGAGAGATTG |
| *COL2* | CAAGAAAGCCCTGCTCATCC | GCAGCCATCCTTCAGAACAG |
| *ACAN* | CTGTCTCCCTCGTGGATACTAC | CTGACACCAATGGTTCCTCTTC |
| *PPARG2* | AACATTTCACAAGAGGTGACCA | CAGCTCTCGGGAATGGGATG |
| *LPL* | TGACAAAGATGCCCAGTGCT | TTTCCCAAAGGACAGAATGC |
| *CBFA1* | CTCCTGTCGGATGCACTTTG | GTGGTCCTGGCTTGGATTAAG |
| *ALP* | TCCCACCACCTCCATCATAC | CAATGGCCAGCACTAAGAAGAG |
| *OC* | TCACACTGCTTGCCCTACT | CCTGCACCTTTGCCAGAATC |
| *TERT* | AAAGCCAGAAACGCAGGGAT | GTCTGTAGTCAGGGCTGGGC |
| *UBC* | GGAGTTTGCCTGCGTTTCT | TCCAGGGTGATGGTCTTACC |
| *Telomere* | GGTTTTTGA[GGGTGA]_4_GGGT | TCCCGACTAT[CCCTAT]_4_CCCTA |
| *36B4* | TGAAGTGCTTGACATCACCGAGGA | CTGCAGACATACGCTGGCAACATT |
| *PAX6* | GGTGAGAAGTGTGGGAACCG | GGCTCGAATATAGGGCTCTGA |
| *Nestin* | CCTCCTAGAGGCGGAGAACT | CAGCTCCAACTTAGGGTCCAA |
| *Brachyury (T)* | GCTGTGAGAGGTATCCTGCC | AGCGTAAGTTGGAGAACTGCT |
| *CXCR4* | ATGGACGGGTTCCGTATATTCA | CCCGGAAACAGGGTTCCTTT |
| *SOX17* | ATGAAGATGAAGGGCGAGGC | GCCTTCCAAGACTTGCCCAG |
| *FOXA2* | GTCTCCCAACCGTCGTTCAG | CACCGCTCCCAGCATACTTT |
| *BRG1* | AGACGAAGTGCCTGACGATGAGAC | GCTTCCGCTTGGGGTTGC |
| *BRM* | GGGACCCCACTGCAGAATAAGC | AATCTGGGGGAGGAGGAAGTTGAG |
| *BAF250A* | AGCCCAGAGATGATGGGTCT | TCCTCCGTGAAGGCCAGATA |
| *BAF155* | GAGGAACTTGACTGGAGATGTG | ATTGCCATCGGTCGACTTT |
| *BAF170* | AGAGTCGACCAACCCCAATG | GTCTGCTGCGGGGTCTTG |
| *BAF60A* | TGAATTTTGTTATGCCACACATGTT | CTCTGCTCGTCTGCTGGTTTC |
| *OCT4* | GCTGACAACAACGAGAATCTGC | ACGCGGACCACATCCTTCTCTAG |
| *SOX2* | GTTCCATGGGCTCAGTGGTCAAG | AAGCGTACCGGGTTTTTCTCCATAC |
| *KLF4* | CCATGGGCCAAACTACCCAC | TGGGGTCAACACCATTCCGT |
| *c-MYC* | CTTACAACACCCGAGCGACA | ATCTTCTTGTTCCTCCTCAGAGT |
| *NANOG* | AGCCCCAGCTCCAGTTTCAGC | AATGATCGTCACATATCTTCAGGCTGTA |
| *LIN28* | CCAAGGGAGACAGGTGCTAC | TGGCAGGGCTATGGATCTCT |
| **Human** | | |
| *OCT4* | CAGGCTCTGAGGTGTGG | GACTCCTGCTTCGCCCTCA |
| *SOX2* | CTCGGACTTGACCACCGAAC | CCGTTCATCGACGAGGCTAA |
| *KLF4* | GGCATGAGCTCTTGGTAATGG | CCAATTCGCTGACCCATCCT |
| *c-MYC* | GCTGGTGCATTTTCGGTTGT | CGTCCTCGGATTCTCTGCTC |
| *NANOG* | TTCTCCTGCCAGTGACTTGG | CTGGGGAAGGCCTTAATGTA |
| *LIN28* | GGTGCACAAAGACATCCACTG | CCTTCCATGTGCAGCTTACTC |
