# Supplementary material for: Epigenetic regulation of BAF60A determines efficiency of miniature swine iPSC generation
Source: Sci Rep. 2022 May 31;12:9039. doi: 10.1038/s41598-022-12919-6 (PMC9156668; doi:10.1038/s41598-022-12919-6)
Supplement: Supplementary file 5 — Supplementary Table S4. [file 41598_2022_12919_MOESM5_ESM.docx]

Table S4 **Detailed information of antibodies used in this study**

| **Primary antibody** | **Company** | **Catalog number** |
| --- | --- | --- |
| FITC, Mouse anti-CD90 | MilliporeSigma | SAB4700706 |
| Alexa Fluor® 647, Mouse anti-CD29 | BD Bioscience | 561496 |
| Alexa Fluor 488, Mouse anti-CD44 | Molecular Probes | A25527 |
| PerCP-Cy^TM^5.5, Mouse anti-CD45 | BD Bioscience | 564105 |
| Mouse anti-CD34 | Life Technology | QE220240 |
| Goat anti-NANOG | Thermo Fisher Scientific | PA5-18406 |
| Rabbit anti-OCT4 | Novus Biologicals | NB100-2379 |
| Rabbit anti-SOX2 | MilliporeSigma | AV38232 |
| Rabbit anti-BAF60A | Thermo Fisher Scientific | A301-595A |
|  | | |
| **Secondary antibody** | **Company** | **Catalog number** |
| Alexa Fluor 647, goat anti-mouse IgG | Thermo Fisher Scientific | A21235 |
| Alexa Fluor 555, donkey anti-Rabbit IgG | Thermo Fisher Scientific | A31572 |
| NL493, donkey anti-goat IgG | R&D | NL003 |
